# A-Prot: protein structure modeling using MSA transformer

**DOI:** 10.1186/s12859-022-04628-8

**Published:** 2022-03-16

**Authors:** Yiyu Hong, Juyong Lee, Junsu Ko

**Affiliations:** 1 Arontier Co, Seoul, Republic of Korea; 2 Department of Chemistry, Division of Chemistry and Biochemistry, Kangwon National University, Chuncheon, Republic of Korea

**Keywords:** Protein structure prediction, Multiple sequence alignment, Protein language model, Deep learning

## Abstract

**Background:**

The accuracy of protein 3D structure prediction has been dramatically improved with the help of advances in deep learning. In the recent CASP14, Deepmind demonstrated that their new version of AlphaFold (AF) produces highly accurate 3D models almost close to experimental structures. The success of AF shows that the multiple sequence alignment of a sequence contains rich evolutionary information, leading to accurate 3D models. Despite the success of AF, only the prediction code is open, and training a similar model requires a vast amount of computational resources. Thus, developing a lighter prediction model is still necessary.

**Results:**

In this study, we propose a new protein 3D structure modeling method, A-Prot, using MSA Transformer, one of the state-of-the-art protein language models. An MSA feature tensor and row attention maps are extracted and converted into 2D residue-residue distance and dihedral angle predictions for a given MSA. We demonstrated that A-Prot predicts long-range contacts better than the existing methods. Additionally, we modeled the 3D structures of the free modeling and hard template-based modeling targets of CASP14. The assessment shows that the A-Prot models are more accurate than most top server groups of CASP14.

**Conclusion:**

These results imply that A-Prot accurately captures the evolutionary and structural information of proteins with relatively low computational cost. Thus, A-Prot can provide a clue for the development of other protein property prediction methods.

**Supplementary Information:**

The online version contains supplementary material available at 10.1186/s12859-022-04628-8.

## Introduction

Modeling the 3D structure of a protein from its sequence has been one of the most critical problems in biophysics and biochemistry [1, 2]. The knowledge of the 3D structure of a protein facilitates the discovery of novel ligands, function annotation, and protein engineering. Due to its importance, the community of computational protein scientists have been developing various prediction methods and assessed their performance in the large-scale blind tests, CASPs, which have continued for over two decades [2]. In CASP14, Deepmind demonstrated that their deep learning-based model, AlphaFold2 (AF2), predicts the 3D structures of proteins from their sequences with extremely high accuracy, comparable to experimental accuracy [3]. The source code of AF2 became publicly available recently, and many model structures of genes of biologically important organisms have been released [3].

The success of AF2 can be attributed to the accurate extraction of coevolutionary information from multiple sequence alignments (MSA). The idea of using coevolutionary information for contact prediction or structure modeling has been widely used [4–12]. However, previous attempts were not successful enough because coevolutionary signals in MSAs were not strong enough or too noisy. Various statistical mechanics-based models were proposed, but their accuracy, discriminating actual contacts from false contacts, was limited. In CASP13, AlphaFold and RaptorX used coevolutionary information from MSAs as input and predicted inter-residue distances [13–15]. They achieved significant improvements than previous CASPs, but their predictions were not accurate enough to obtain model structures comparable to experiments. Finally, in CASP14, with the help of the attention algorithm, truly accurate extraction of actual residue-residue contact signals from MSAs becomes available [3].

Despite this remarkable achievement of AF2, certain limitations remain for this technology to be widely accessible and used for further development. First, the source code for training AF2 is not open-sourced yet. In the first release of AF2, only the production, model generation, part of AF2, and the model parameters are open. Second, training the AF2 architecture is computationally expensive. It is reported that Deepmind used 128 TPUv3 cores for approximately one week and four more days to train AF2. This amount of computational resources are not readily accessible for most academic groups. Thus, developing lighter and general models is still necessary.

To extract evolutionary information from MSAs, various protein language models have been proposed [16–22]. Rao et al. proposed the MSA transformer model, an unsupervised protein language model using the MSA of a query sequence instead of a single query sequence. The model uses row and column attention of input MSAs and masked language modeling objectives. It is demonstrated that the model successfully predicted long-range contacts between residues. In addition, the model predicted other properties of proteins, such as secondary structure prediction and mutational effects, with high accuracy [20]. These results indicate that the MSA Transformer model extracts the characteristics of proteins from their MSA profiles efficiently.

This study developed a new protein 3D structure prediction method, A-Prot, using MSA Transformer [22]. For a given MSA, we extracted evolutionary information with MSA Transformer. The extracted row attention map and input features were converted to a 2D residue-residue distance map and dihedral angle predictions. We benchmarked the 3D protein structure modeling performance using the FM/TBM-hard targets of CASP13 and 14 [23, 24]. The results show that A-Prot outperforms most top server groups of CASP13 and 14 in terms of long-range contact predictions and 3D protein structure modeling.

## Methods

### Overview

**Fig. 1:**
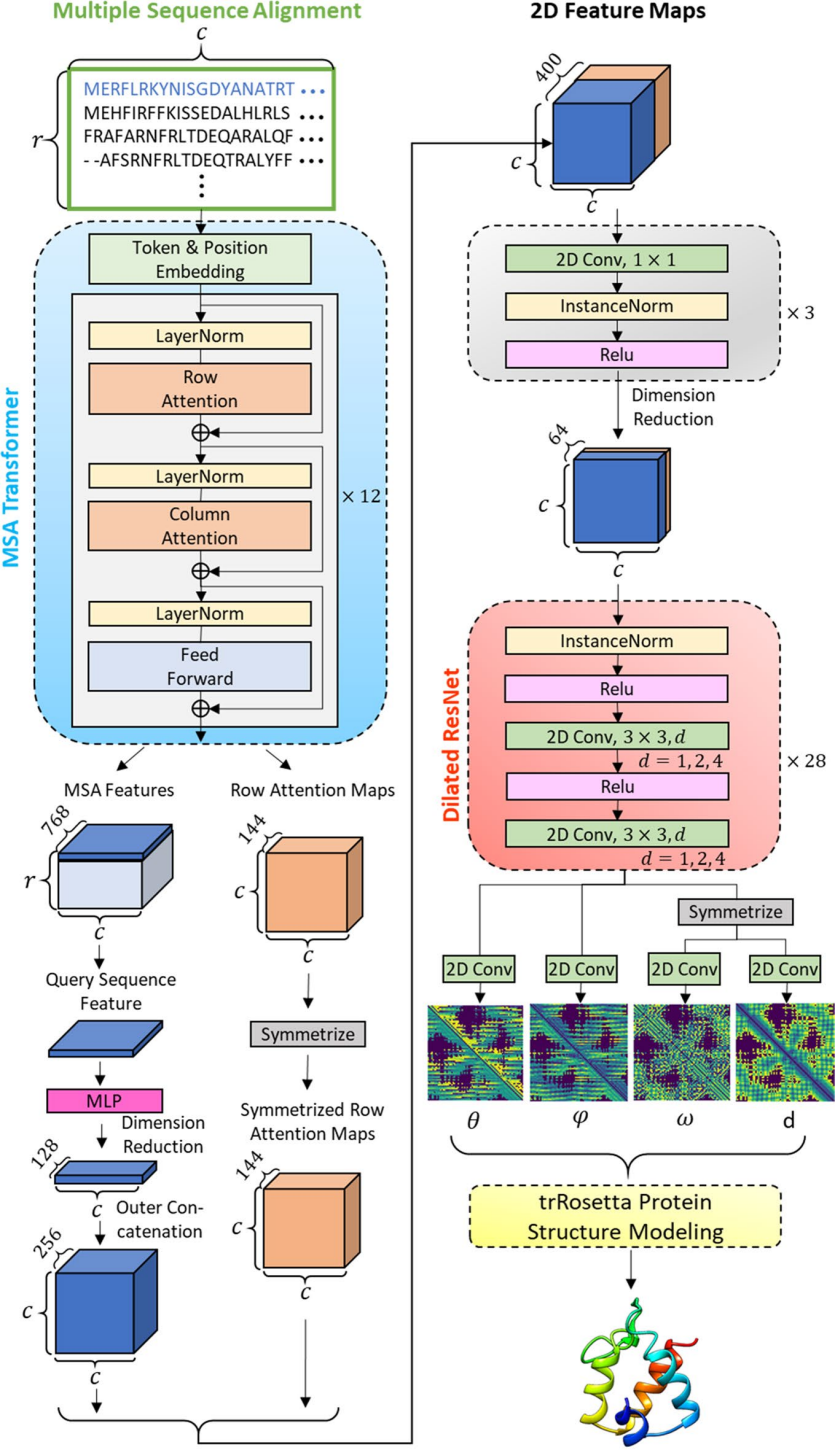
Pipeline of the proposed protein 3D structure prediction method. Input an MSA to the MSA Transformer to extract MSA Features and row attention maps. Then, the MSA features corresponding to the query sequence and the row attention maps are combined to a 2D feature maps by a set of transformations. Next, the 2D feature maps are input to a Dilated ResNet after dimension reduction to output inter-residue geometries, which further input to the trRosetta Protein Structure Modeling to output a predicted protein 3D structure

The overall pipeline of the proposed protein 3D structure prediction method is shown in Fig. 1. We mainly combine the works of the MSA Transformer [22] and the trRosetta [25]. Given an MSA, it will input to the MSA Transformer to output MSA features and row attention maps. After a series of transformations like dimension reduction and concatenation etc., these two kinds of features will be transformed and combined into 2D feature maps, which is suitable for input to the trRosetta to finally output protein 3D structure.

The MSA Transformer used in this paper was an already pre-trained version that was learned from 26 million MSAs. It plays a role as a feature extractor that inputs an MSA and outputs its relative features.

trRosetta consists of a deep neural network part and protein structure modeling part. The deep neural network is modified to receive the MSA features from the MSA Transformer instead of the original manually engineered features generated by the statistic approach. Nevertheless, the protein structure modeling part remains the same.

### Dataset and MSA generation

We used the procedure with MSA transformer to generate the MSAs of target sequences (Rao, Liu, et al., 2021). The MSA of a query sequence was generated using Jackhammer [26], HHblits ver. 3.3.0, and HHsearch [27] together with the unicluster30_2017_10 [28] and BFD [29] databases. If not specified, all training and test MSAs are used BFD. If the number of detected homologous sequences exceeds 256, up to 256 sequences were selected through diversity minimization [22]. The upper limit of the number of sequences was determined by the maximum size of GPU memory of the NVIDIA Quadro RTX 8000 GPU (48 GB) card.

To perform contact predictions, a customized non-redundant protein structure database is compiled, and we named it PDB30. The PDB30 dataset consists of protein structures deposited in PDB before Apr-30–2018 whose resolution is higher than 2.5 A and sequence length is longer than 40 amino acids. Using 102,300 sequences that satisfy the condition, clustering analysis was performed using MMSeq2 [30] with a threshold of sequence identity of 30%, leading to 16,612 non-redundant sequences.

### Network architecture

Let us define an input MSA as an $r \times c$ character matrix, where $r$ and $c$ are rows (number of sequences) and columns (sequence length) in the MSA, respectively. Through the token and position embedding of the MSA Transformer, the input MSA is embedded into a $r \times c \times 768$ tensor which is the input and output of each attention block. The attention block is composed in the order of row attention layer, column attention layer, and feed-forward layer. A layer normalization operation is followed by each layer. And each attention layer has 12 attention heads. The MSA Transformer is a stack of 12 such attention blocks.

Two kinds of features were extracted from the MSA Transformer to construct 2D feature maps by a series of transformations. (1) One is the last attention block’s output, a $r \times c \times 768$ tensor; we named it MSA features (Fig. 1). Only features corresponding to the query sequence are selected, which is a $1 \times c \times 768$ tensor. Then, the dimension of this feature is reduced to 128 by an MLP (multi-layer perceptron) consist of 3 linear layers with neuron sizes 384, 192, 128. The dimension reduced 1D feature is then outer concatenated (redundantly expanding horizontally and vertically and then stacked together) to form a query sequence feature of a $c \times c \times 256$ tensor. (2) The other one is row attention maps that are derived from each attention head of each row attention layer, totally $12 \times 12 = 144$ attention maps stacked to shape in $c \times c \times 144$. Then it is symmetrized by adding it to its transposed tensor to yield symmetrized row attention maps. The query sequence feature and the symmetrized row attention maps are concatenated in the feature map dimension to form a 2D feature map that is a $c \times c \times 400$ tensor.

The dimension of the 2D feature maps afterward reduced to 64 by three convolutional layers that consist of 256, 128, 64 kernels of size $1 \times 1$ [31], where each convolutional layer is followed by an instance normalization and a ReLU activation. Then this $c \times c \times 64$ tensor is input to the Dilated ResNet consist of 28 residual blocks [32], each having one instance normalization, two ReLU activations, and two convolutional layers each with 64 kernels of size $3 \times 3$. Dilation is applied to the convolutional layers cycle through the residual blocks with rates of 1, 2, 4. After the last residual block, there are four independent convolutional layers with 25, 13, 25, 37 kernels of size $1 \times 1$, each for predicting inter-residue geometries of $\theta ,{ }\varphi ,{ }\omega ,{\text{ d}}$, respectively. Please refer to the reference [25] for more details about the inter-residue geometries as we used the same settings. Finally, the trRosetta Protein Structure Modeling module will predict and modeling the protein 3D structure based on the inter-residue geometries information.

### Training and inference

At the training stage, we fixed the parameters in the MSA Transformer. In contrast, parameters in the other deep neural networks were trained with a batch size of 16 with gradient accumulation steps, a learning rate of 1e − 3, using the RAdam optimizer [33], the categorical cross-entropy loss was calculated with equal weight for the four inter-residue geometry objectives [25]. The ground truth values for the inter-residue geometries are all discretized into bins which have same number as corresponding convolutional kernels ($\theta$ for 25 bins, $\varphi$ for 13 bins, $\omega$ for 25 bins, ${\text{d}}$ for 37 bins); each bin is treated as a classification label. The total model was trained end-to-end on an NVIDIA Quadro RTX 8000 GPU (48 GB) for around 40 epochs which took about five days.

An MSA subsampling strategy is applied during training, not only for regarding as data augmentation to train a robust model, but also for preventing the GPU from running out of memory when filled with large MSA. We randomly select MSA rows, up to a maximum of $2^{14} /c$, and down to a minimum of 16, though always including the query sequences in the first row. Large proteins of more than 1023 residues long were discarded during training. We subsampled MSA with 256 sequences at the inference stage by adding the sequence with the lowest average hamming distance. We performed trRosetta protein structure modeling five times with the same input and selected the structure with the lowest energy for protein structure similarity measurement.

## Results and discussion

### Benchmarking contact prediction

First, we benchmarked the long-range contact prediction performance of A-Prot using the FM and FM/TBM targets of CASP13 [24]. The benchmark results show that the performance of our model outperforms that of the existing methods (Table 1). We compared the precision of our model's top L/5, L/2, long-range contact predictions (long-range: sequence separation of the residue pair ≥ 24) and the other existing methods. The performance measures of the other methods are adopted from the reference [34]. The top L/5, Top L/2, and L contact precisions of our model are 0.812, 0.710, and 0.562, which are higher than those of the other methods. For example, DeepDist, one of the state-of-the-art methods, predicted the top L/5, L/2, and L with precisions of 0.793, 0.661, and 0.517. Also, compared with AlphaFold predictions of CASP13, A-Prot predicted more accurately in all three measures by 7–9%.

**Table 1:** Contact Precision on CASP13 FM and FM/TBM targets corresponding to 43 domains (results are adapted from DeepDist [34])

Group	Top L	Top L/2	Top L/5
TripletRes	0.451	0.587	0.700
AlphaFold	0.497	0.629	0.742
RaptorX-Contact	0.481	0.612	0.744
trRosetta	0.506	0.652	0.751
DeepDist	0.517	0.661	0.793
A-Prot (w BFD)	**0.562**	**0.710**	**0.812**
A-Prot (w/o BFD)	0.540	0.681	0.780

The highest score of each column is highlighted in bold

We also compared the contact prediction accuracy of A-Prot with MSA-Transformer, which is the baseline of A-Prot. The performance of long-range Top L and Top L/5 for supervised contact prediction on CASP13 FM targets were 0.546 and 0.775 respectively (Rao, Meier, et al., 2021). The contact precision of Top L and Top L/5 for A-prot were 0.539 and 0.785, which is comparable to those of MSA Transformer (Additional file 1: Table S2). We also investigated the effect of a MSA to contact prediction by predicting structures with the MSAs obtained without BFD and obtained with DeepMSA [35]. The assessment shows that not using BFD deteriorates the quality of models. The accuracies of Top L and Top L/5 contacts decreased by 2.5% and 4.0%, respectively. When DeepMSA was used, the Top L and Top L/5 accuracies dropped by 3.9% and 2.0%, respectively. These results show that considering a gigantic meta-genome DB helps improve prediction quality, but not significantly.

To investigate how the number of residual blocks affects the prediction quality, ablation tests were performed by changing the number of the residual blocks (Additional file 1: Table S4). We performed contact and structure prediction with 4, 16, 28, and 40 residual blocks. The contact prediction results show that A-Prot calculations with 28 blocks resulted in the best contact prediction. Interestingly, using more blocks, 40, decreased contact prediction accuracy. Fewer residual blocks, 4 and 16, led to significantly worse contact prediction results. In terms of model accuracy, A-Prot with 40 blocks resulted in the highest TMscore although contact predictions were most accurate with 28 blocks. These results show that A-Prot with 28 blocks is close to the optimal model considering both the prediction accuracy and computational cost.

### Benchmark on model accuracy

In addition to contact prediction, we also compared the quality of protein models predicted by A-Prot with those submitted by the top-performing server groups of CASP14 (Table 2). The highest score of each column is highlighted in bold. First, we modeled the structures of 25 FM/TBM and TBM-hard targets of CASP14. The average TM-score and lDDT score of the models were compared with those of the following server groups: FEIG-S [36], BAKER-ROSETTASERVER [37], Zhang-Server, and QUARK [38]. The model structures of the other groups were downloaded from the archive of the CASP14 website, and TM-score and lDDT scores were recalculated with the crystal structures and domain information for a fair comparison.

**Table 2:** The average TM-score and lDDT of the model structures of 25 CASP14 FM, FM/TBM, and TBM-hard domains

Server group	TM-score	*P*-value (TM-score)	lDDT	*P*-value (lDDT)
FEIG-S	0.461	0.0007	0.413	0.0172
BAKER-ROSETTASERVER	0.517	0.0391	0.455	0.2636
Zhang-Server	**0.595**	0.6684	0.489	0.8510
QUARK	0.588	0.6208	0.484	0.7408
A-Prot	0.576	**–**	**0.499**	**–**

A-Prot outperforms the other top server groups in terms of lDDT. Compared with BAKER- ROSETTASERVER and FEIG-S, A-Prot models are consistently more accurate in both measures. The P-values show that A-Prot outperforms ROSETTASERVER and FEIG-S statistically significantly. These results show that the accuracy of A-Prot is comparable to or better than the top-performing server groups that participated in CASP14 [23]. In terms of TM-score, A-Prot is showing slightly worse results, 0.576, than Zhang-Server and QUARK, whose TM-scores are 0.595 and 0.588. However, the P-values show that the A-Prot results are not meaningfully different.

We also compared the performance of A-Prot with trRosetta [25] by modeling structures using trRosetta with the identical MSAs that we used for A-Prot (Table 3). The results show that A-Prot significantly outperforms trRosetta using the identical MSAs. In terms of dihedral angle predictions, A-Prot improved the correlation coefficient between ground truth and predictions by 0.071 in average. In addition, A-Prot generated better models with higher TMscore and lDDT values than the trRosetta results. The average TMscore and lDDT enhanced by 0.048 and 0.042 respectively.

**Table 3:** Performance of A-Prot and trRosetta using same MSA of ours on 25 CASP14 FM, FM/TBM, TBM-Hard domains. (Inter-residue distance and angles are measured using Pearson correlation between predicted bin indexes of max probability and ground truth, Top L for long range contact precision)

Method	$\theta$	$\varphi$	$\omega$	${\text{d}}$	Top L	TMS	lDDT
trRosetta	0.539	0.498	0.459	0.362	0.363	0.524	0.457
A-Prot	0.604	0.578	0.528	0.464	0.424	0.576	0.499

In contrast to MSA-Transformer or ROSETTA, A-Prot used the subsampling strategy that minimizes the diversity of sequences to reduce the sizes of MSAs. For protein language models, four subsampling strategies have been tested: random, diversity maximization, diversity minimization, and HHFilter to reduce the size of input MSAs while preserving prediction accuracies. It was shown that diversity maximizing performed best for the supervised contact prediction [22]. On the contrary, trRosetta generates a MSA in a similar manner to the diversity minimizing approach [25]. During the development of A-Prot, we tried both diversity minimization and maximization approaches. The prediction results show that diversity minimizing yields more accurate models than diversity maximization (Additional file 1: Table S3). We predicted the structures of 43 domains of CASP13 FM, FM/TBM and 25 domains of CASP14 FM, FM/TBM and TBM-hard targets using MSAs subsampled with diversity minimization and maximization. In average, the models generated with the MSAs obtained with diversity minimization have higher TM-scores, 0.658 and 0.555 for the CASP13 and CASP14 targets, than those with diversity maximization, 0.603 and 0.532. Thus, we employed the diversity minimization approach for A-Prot.

### A head-to-head comparison with ROSETTASERVER

**Fig. 2:**
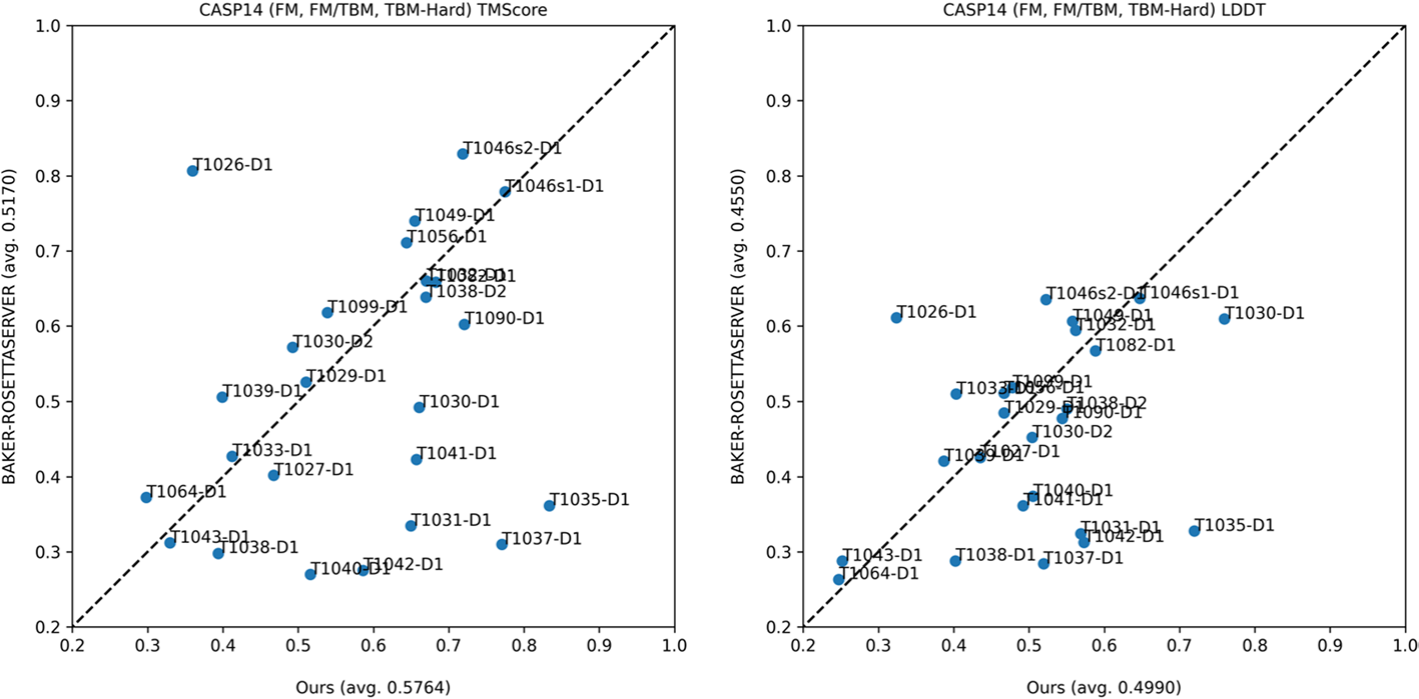
TM-score and lDDT on CASP14 FM, FM/TBM and TBM-hard 25 domains compared with BAKER-ROSETTASERVER

**Fig. 3:**
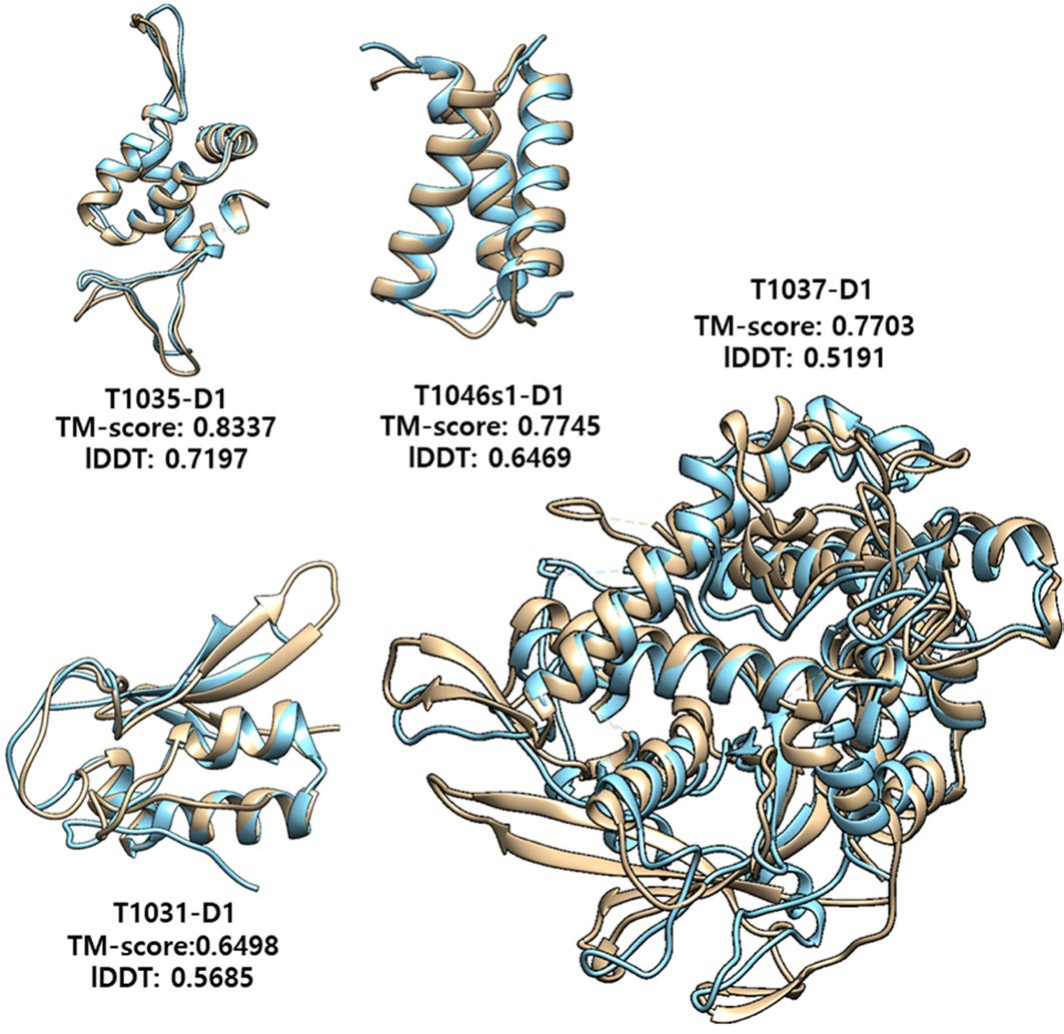
Model comparison of four high-quality CASP14 models generated from our method versus their native structures. Brown: native structure; Blue: model

Because A-Prot uses trRosetta for modeling structure at the final stage, we performed a head-to-head comparison of A-Prot models with the ROSETTASERVER models to identify improvement in residue-residue distance predictions (Fig. 2). In terms of TM-score, many predictions made by A-Prot are significantly better than BAKER-ROSETTASERVER. For instance, the model qualities of five targets that were predicted to have TM-score less than 0.4 by ROSETTASERVER were improved higher than 0.4, corresponding to a correct fold prediction. Four highly accurate models are depicted in Fig. 3.

Similarly, in terms of the lDDT measure, prediction results of eight targets were improved significantly. On the contrary, only two targets deteriorated more than 0.05. Therefore, A-Prot predicts residue-residue distances and dihedral angles better than most server groups participated in CASP14 [2].

On the other hand, nearly all worse predictions by A-Prot deteriorated only by a small margin except the T1026-D1 target. For T1026-D1, A-Prot did not predict its correct fold. This incorrect prediction is attributed to an incomplete MSA. T1026-D1 is a protein consisting of a virus capsid (PDB ID: 6S44). Our sequence search procedure found only less than 30 sequences, which appear to be not enough to extract correct evolutionary information. When T1026-D1 is modelled with the MSA of trRosetta containing more than 100 sequences, T1026-D1, a model with a similar TM-score is obtained. Thus, the failure of T1026-D1 suggests that having enough homologous sequences is critical in accurate 3D structure modeling using MSA-Transformer. In other words, a more extensive sequence search may improve the model accuracy of A-Pro. Except for T1026-D1, the deviations of all worse predictions than ROSETTASERVER were less than 0.1 TM-score, much smaller than the improvements.

## Conclusion

In this study, we introduced a new protein structure prediction method, A-Prot, using MSA Transformer. Our benchmark results on the CASP13 TBM/FM and FM targets show that A-Prot predicts long-range residue-residue contacts more accurately than the existing methods. We also assessed the quality of protein structure models based on the predicted residue-residue distance information. The model generated by A-Prot is more accurate than most of the server groups that participated in CASP14. The average lDDT of A-Prot models is higher than that of all server group models. In terms of TM-score, our model is slightly worse than QUARK and Zhang-Server. These results show that our approach yields highly accurate residue-residue distance predictions.

A-Prot requires less computational resources than the other state-of-the-art protein structure prediction models [2]. The source code of AlphaFold2 is only partially open [3]. Its model parameters are fixed, and only the structure modeling part is open. Thus, it is hard to tune AlphaFold2 for bespoken purposes. In addition, training the AlphaFold2 architecture requires a significant amount of computational resources, which is not assessable for most academic groups. On the other hand, A-Prot can be trained with a single GPU card. In summary, A-Prot will open new possibilities for training novel deep-learning-based models to predict various properties of proteins only using sequence information.

## Supplementary Information

**Additional file 1**. The additional analysis on the modeling results are provided.

## Data Availability

All predicted model structures and the multiple sequences alignments used as the inputs for the A-Prot models are available at https://github.com/arontier/A_Prot_Paper.

## Abbreviations

MSA: Multiple sequence alignment
FM: Free modeling
TBM: Template-based modeling
RMSD: Root mean square deviation
