# Supplementary material for: A-Prot: protein structure modeling using MSA transformer
Source: BMC Bioinformatics. 2022 Mar 16;23:93. doi: 10.1186/s12859-022-04628-8 (PMC8925138; doi:10.1186/s12859-022-04628-8)
Supplement: Supplementary file 1 — Additional file 1. The additional analysis on the modeling results are provided. [file 12859_2022_4628_MOESM1_ESM.docx]

Table. S1. CASP13 FM, FM/TBM 43 domains (35 targets) and CASP14 FM, FM/TBM, TBM-Hard 25 domains (23 targets) that used for evaluation in this paper.

| CASP13 FM, FM/TBM domain list (FM domains marked in bold) | | | | |
| --- | --- | --- | --- | --- |
| T0949-D1 | **T0950-D1** | T0953s2-D1 | **T0953s2-D2** | **T0953s2-D3** |
| **T0957s1-D1** | **T0957s2-D1** | T0958-D1 | **T0960-D2** | **T0963-D2** |
| **T0968s1-D1** | **T0968s2-D1** | **T0969-D1** | T0970-D1 | **T0975-D1** |
| T0978-D1 | **T0980s1-D1** | **T0981-D2** | T0981-D3 | T0986s1-D1 |
| **T0986s2-D1** | **T0987-D1** | **T0987-D2** | **T0989-D1** | **T0989-D2** |
| **T0990-D1** | **T0990-D2** | **T0990-D3** | **T0991-D1** | T0992-D1 |
| T0997-D1 | **T0998-D1** | **T1000-D2** | **T1001-D1** | T1005-D1 |
| T1008-D1 | **T1010-D1** | **T1015s1-D1** | **T1017s2-D1** | T1019s1-D1 |
| **T1021s3-D1** | **T1021s3-D2** | **T1022s1-D1** |  |  |
| CASP14 FM, FM/TBM, TBM-Hard domain list | | | | |
| T1026-D1 | T1027-D1 | T1029-D1 | T1030-D1 | T1030-D2 |
| T1031-D1 | T1032-D1 | T1033-D1 | T1035-D1 | T1037-D1 |
| T1038-D1 | T1038-D2 | T1039-D1 | T1040-D1 | T1041-D1 |
| T1042-D1 | T1043-D1 | T1046s1-D1 | T1046s2-D1 | T1049-D1 |
| T1056-D1 | T1064-D1 | T1082-D1 | T1090-D1 | T1099-D1 |

Table. S2. Supervised learning based model contact precision (long-range) on CASP13 FM 31 domains.

|  | Top L | Top L/5 |
| --- | --- | --- |
| MSA Transformer  (2021.1) | 0.571 | 0.861 |
| MSA Transformer  (2021.7) | 0.546 | 0.775 |
| A-Prot (w BFD) | 0.539 | 0.785 |
| A-Prot (w/o BFD) | 0.514 | 0.745 |
| A-Prot (w DeepMSA) | 0.475 | 0.725 |

Table. S3. TMScore of A-Prot on CASP13 and CASP14 using diversity maximizing and minimizing MSA subsampling strategies (middle results of A-Prot).

| MSA subsampling method | CASP13 FM, FM/TBM  (43 domains) | CASP14 FM, FM/TBM, TBM-Hard  (25 domains) |
| --- | --- | --- |
| Diversity maximizing | 0.603 | 0.532 |
| Diversity minimizing | 0.658 | 0.555 |

Table. S4. Ablation study for number of residual blocks used in A-Prot, using CASP14 FM, FM/TBM, TBM-Hard 25 domains for evaluation. (Long-range contact precision for Top L, L/2, L/5)

| Number of residual blocks | Top L | Top L/2 | Top L/5 | TMS | lDDT |
| --- | --- | --- | --- | --- | --- |
| 4 | 0.372 | 0.487 | 0.636 | 0.563 | 0.477 |
| 16 | 0.404 | 0.530 | 0.640 | 0.558 | 0.495 |
| 28 | 0.425 | 0.555 | 0.684 | 0.576 | 0.499 |
| 40 | 0.412 | 0.542 | 0.659 | 0.581 | 0.492 |

Table. S5. A-Prot and trRosetta running time on 23 CASP14 targets. (except structure modeling part)

| Method | Total time (average time) |
| --- | --- |
| trRosetta | 12.42 seconds (0.54 seconds) |
| A-Prot | 13.53 seconds (0.59 seconds) |

Table. S6. A-Prot performance of short-, medium-, long range contact precision on CASP13 and CASP14 datasets.

| Dataset | Short-range | | | Medium-range | | | Long-range | | |
| --- | --- | --- | --- | --- | --- | --- | --- | --- | --- |
|  | Top L | Top L/2 | Top L/5 | Top L | Top L/2 | Top L/5 | Top L | Top L/2 | Top L/5 |
| CASP13 FM  (w/o BFD) | 0.272 | 0.450 | 0.710 | 0.401 | 0.581 | 0.782 | 0.514 | 0.657 | 0.745 |
| CASP13  FM, FM/TBM  (w/o BFD) | 0.291 | 0.472 | 0.729 | 0.401 | 0.591 | 0.800 | 0.540 | 0.681 | 0.780 |
| CASP14 FM, FM/TBM, TBM-Hard  (w BFD) | 0.204 | 0.349 | 0.549 | 0.248 | 0.389 | 0.581 | 0.425 | 0.555 | 0.684 |
